# Successful transcatheter closure of a congenital aorto-right ventricular fistula after Norwood procedure

**DOI:** 10.1016/j.xjtc.2026.102199

**Published:** 2026-01-19

**Authors:** Eijiro Yamashita, Sreejith Mylavarapu, Rohit S. Loomba, Alan Nugent, Pei-Ni Jone, Allison B. Davila, Elisabeth Martin, Michael C. Mongé, David S. Winlaw

**Affiliations:** a Heart Center, Ann & Robert H. Lurie Children's Hospital of Chicago, Chicago, Ill; b Pediatric Cardiovascular-Thoracic Surgery, Northwestern University Feinberg School of Medicine, Chicago, Ill; c Pediatric Cardiology, Northwestern University Feinberg School of Medicine, Chicago, Ill

**Figure:**
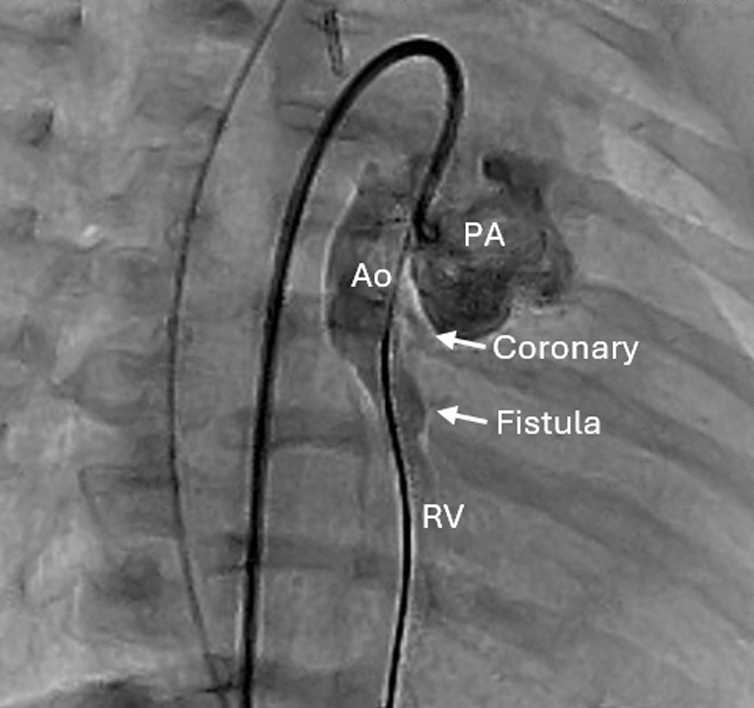
Aorta to RV fistula evident on angiography after Norwood operation.

**Central Message**

A coexisting Ao-RV fistula in a patient with HLHS is rare. Early catheterization enables prompt diagnosis, and transcatheter closure can avoid high-risk reoperation after Norwood procedure.

Hypoplastic left heart syndrome (HLHS) is a severe congenital heart defect requiring staged palliative surgeries, beginning with the Norwood procedure. Postoperative complications such as shunt malfunction, neoaortic obstruction, and tricuspid insufficiency can cause hemodynamic instability. The presence of an aorto-right ventricular (Ao-RV) fistula is exceptionally rare and poses unique diagnostic and management challenges in the postoperative period. We report a neonate with HLHS who underwent the challenging diagnosis and successful transcatheter closure of an Ao-RV fistula after Norwood, highlighting the value of early catheterization in identifying occult lesions that result in hemodynamic compromise after the Norwood procedure. Institutional review board approval was not required, and the need for informed consent was waived because of the retrospective and deidentified nature of this case (“Not Human Research” determination received August 15, 2025, Institutional review board ID# STUDY00000545).

## Case Presentation

**Figure 1:**
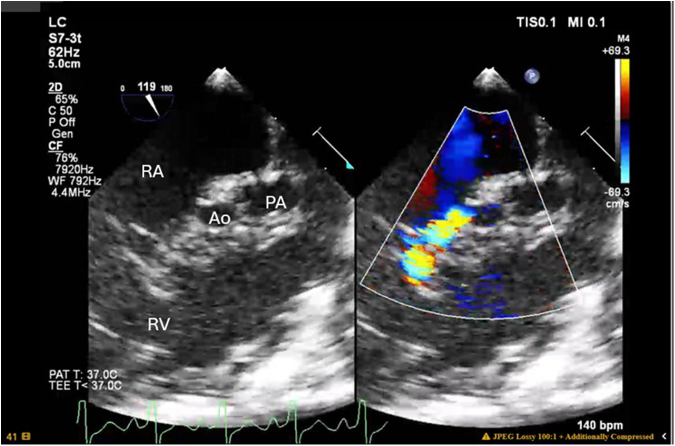
Intraoperative transesophageal echocardiogram demonstrating shunt flow from the aorta to right atrium/right ventricle. *RA*, Right atrium; *Ao*, aorta; *PA*, pulmonary artery; *RV*, right ventricle.

A full-term male neonate with HLHS (mitral and aortic atresia) underwent a Norwood procedure and RV-to-pulmonary artery shunt with valved conduit using femoral vein homograft and expanded polytetrafluoroethylene conduit on day 3 of life. Preoperative transesophageal echocardiogram (TEE) suggested flow from the native aortic root to the right atrium, but anatomy could not be clearly delineated. There were no signs of coronary ischemia or RV dysfunction. Intraoperative TEE taken after repair demonstrated unchanged RV function, mild tricuspid regurgitation, an unobstructed aortic arch, and a patent RV-to-pulmonary artery shunt. The previously noted flow into the right atrium remained, but its precise origin could not be clearly visualized (Figure 1).

**Figure 2:**
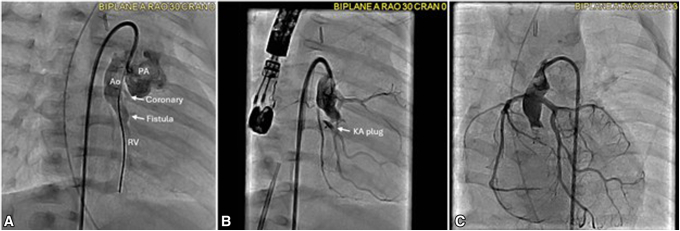
A, Preocclusion aortic root angiogram fistula flow into the RV. B, Postocclusion aortic root angiogram showing cessation of fistula flow and preserved coronaries. C, Follow-up angiogram 3 months after occlusion is shown, confirming unobstructed coronaries and no residual fistula flow. *RV*, Right ventricle; *Ao*, aorta; *PA*, pulmonary artery; *KA*, KA Medical.

Postoperatively, the patient developed congestive heart failure, likely as the result of volume overload augmented by the fistula. Given limited clinical progress and ongoing hemodynamic concerns, cardiac catheterization was performed. Aortic root angiography demonstrated a fistula originating from the native aortic root and draining directly into the RV, confirming the presence of an Ao-RV fistula (Figure 2, *A*). Although coronary arteries were normal in origin and course, the fistula's proximity raised concern for coronary steal. After multidisciplinary discussion, transcatheter closure was chosen as a result of the lesion's accessibility and the risks of reoperation. Under TEE guidance, a 5-mm plug (KA Medical) was advanced into the RV and carefully pulled back to rest just at the entry point from the tunnel to the RV. Final angiography demonstrated minimal residual shunt and no interference with coronary flow (Figure 2, *B*, Video 1). Hemodynamics stabilized rapidly postintervention. Follow-up angiogram 3 months after fistula closure exhibited unobstructed coronary flow and no residual fistula flow (Figure 2, *C*). The patient subsequently underwent bidirectional Glenn palliation and remains clinically stable at 6 months of age.

## Discussion

In this case, preoperative transthoracic echocardiogram did not clearly identify a fistula connection; the possibility of a fistula was only recognized by TEE in the operating room after anesthesia induction. Even at that time, however, the fistula's full course could not be completely delineated. In the postoperative angiography, contrast injected into the aorta opacified both the coronary arteries and the fistula, suggesting that the coronary arteries would have received sufficient cardioplegia during the procedure. Congenital Ao-RV fistulas are exceedingly rare. The aortic outflow tract normally develops from 3 segments—proximal, intermediate, and distal—with the fusion of their cushions forming the arterial roots; failure of this process can create abnormal channels such as Ao–RV fistulas.^1^ To our knowledge, percutaneous closure of iatrogenic Ao–RV fistulas has been reported in an adult case after transcatheter aortic valve implantation,^2^ but never in a pediatric patient. A 5-mm plug (KA Medical) with 3 short discs was selected to close the fistula. This device configuration allowed retrograde delivery through a catheter. One disc was deployed in the RV, and the remaining 2 discs were positioned within the tunnel to seal the fistula. Intraprocedure TEE confirmed that tricuspid valve function was not compromised. The patient received rivaroxaban (Xarelto) and aspirin; pre-bidirectional Glenn catheterization 3 months later showed no thrombosis, consistent with expected device endothelialization. Prompt angiography identified the fistula as the cause of hemodynamic compromise. Catheter-based closure minimized circulatory disruption, allowed precise occlusion without affecting coronary flow, and resulted in marked clinical improvement; subsequently, the patient successfully underwent bidirectional Glenn.

## Conflict of Interest Statement

Dr Jone is a consultant for Philips and GE. All other authors reported no conflicts of interest.

The *Journal* policy requires editors and reviewers to disclose conflicts of interest and to decline handling or reviewing manuscripts for which they may have a conflict of interest. The editors and reviewers of this article have no conflicts of interest.

## Supplementary Data

Video 1 Aortic root angiogram before occlusion device deployment. Video available at: https://www.jtcvs.org/article/S2666-2507(26)00006-4/fulltext.
