# Supplementary figures and images for: Successful transcatheter closure of a congenital aorto-right ventricular fistula after Norwood procedure
Source: JTCVS Tech. 2026 Jan 19;36:102199. doi: 10.1016/j.xjtc.2026.102199 (PMC13069542; doi:10.1016/j.xjtc.2026.102199)

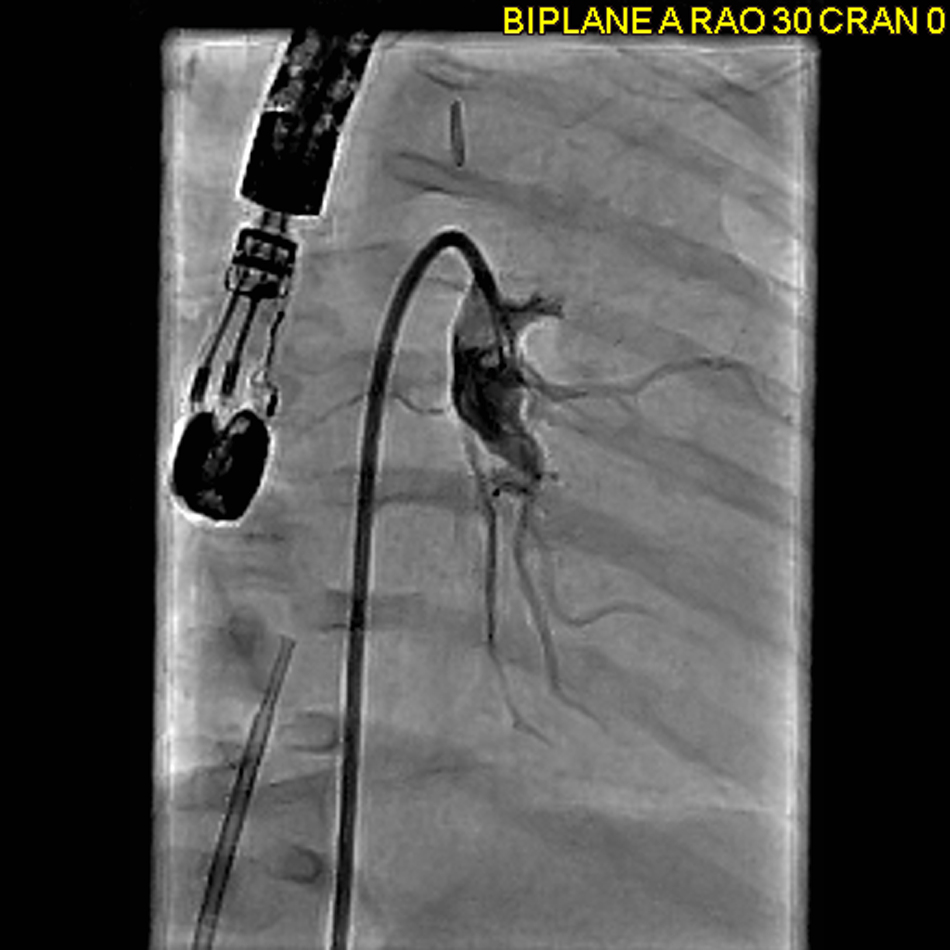

Supplement: Video 1 — Aortic root angiogram before occlusion device deployment. Video available at: https://www.jtcvs.org/article/S2666-2507(26)00006-4/fulltext. [file fx2.jpg]
